# The VHL-dependent regulation of microRNAs in renal cancer

**DOI:** 10.1186/1741-7015-8-64

**Published:** 2010-10-21

**Authors:** Calida S Neal, Michael Z Michael, Lesley H Rawlings, Mark B Van der Hoek, Jonathan M Gleadle

**Affiliations:** 1 Renal Unit, School of Medicine, Flinders University, Flinders Medical Centre, Adelaide, Australia; 2 Department of Gastroenterology, School of Medicine, Flinders University, Flinders Medical Centre, Adelaide, Australia; 3 Familial Cancer Section, Department of Genetics and Molecular Pathology, IMVS, Adelaide, Australia; 4 Adelaide Microarray Centre, School of Molecular and Biomedical Sciences, University of Adelaide, Adelaide, Australia

## Abstract

**Background:**

The commonest histological type of renal cancer, clear cell renal cell carcinoma (cc RCC), is associated with genetic and epigenetic changes in the von Hippel-Lindau (VHL) tumour suppressor. VHL inactivation leads to induction of hypoxia-inducible factors (HIFs) and a hypoxic pattern of gene expression. Differential levels of specific microRNAs (miRNAs) are observed in several tumours when compared to normal tissue. Given the central role of VHL in renal cancer formation, we examined the VHL-dependent regulation of miRNAs in renal cancer.

**Methods:**

VHL-dependent miRNA expression in cc RCC was determined by microarray analysis of renal cell line RCC4 with mutated VHL (RCC4-VHL) and reintroduced wild-type VHL (RCC4 + VHL). Five miRNAs highly upregulated in RCC4 + VHL and five miRNAs highly downregulated in RCC4 + VHL were studied further, in addition to miR-210, which is regulated by the HIF-VHL system. miRNA expression was also measured in 31 cc RCC tumours compared to adjacent normal tissue.

**Results:**

A significant increase in miR-210, miR-155 and miR-21 expression was observed in the tumour tissue. miR-210 levels also showed a correlation with a HIF-regulated mRNA, carbonic anhydrase IX (CAIX), and with VHL mutation or promoter methylation. An inverse correlation was observed between miR-210 expression and patient survival, and a putative target of miR-210, iron-sulfur cluster assembly protein (ISCU1/2), shows reciprocal levels of mRNA expression in the tumours.

**Conclusions:**

We have identified VHL-regulated miRNAs and found that for some the regulation is HIF-dependent and for others it is HIF-independent. This pattern of regulation was also seen in renal cancer tissue for several of these miRNAs (miR-210, miR-155, let-7i and members of the miR-17-92 cluster) when compared with normal tissue. miR-210 showed marked increases in expression in renal cancer and levels correlated with patient survival. The inverse correlation between miR-210 levels and ISCU1/2 provides support for the hypothesis that ISCU1/2 is a target of miR-210 and that it may contribute to the anaerobic respiration seen in renal (and other) tumours.

See Commentary: http://www.biomedcentral.com/1741-7015/8/65

## Background

Renal cell carcinoma accounts for 2-3% of malignant diseases in adults, with an increasing worldwide incidence of over 200,000 new cases and 100,000 deaths per year [1]. The understanding of the pathogenesis of the commonest histological type, clear cell renal cell carcinoma (cc RCC), has been considerably advanced by the recognition that the gene encoding the von Hippel-Lindau (VHL) protein is defective in patients with VHL disease and in the majority of sporadic cc RCCs resulting in activation of a hypoxic pattern of gene expression [2,3]. The exposure of cells to hypoxia normally leads to the coordinated regulation of many genes by the transcription factor hypoxia-inducible factor (HIF). In this process, the VHL protein functions as an E3 ubiquitin ligase which recognises and binds to two specific hydroxyprolyl residues in HIF-1α and HIF-2α and facilitates ubiquitination, leading to rapid proteasomal degradation [4,5]. In the presence of oxygen, HIF-α molecules undergo prolyl hydroxylation catalysed by three homologous 2-oxoglutarate-dependent dioxygenases, PHD1, PHD2 and PHD3 [6]. The protein products of this broad array of hypoxically regulated genes have critical roles in processes such as energy metabolism, angiogenesis, growth and apoptosis [5,7,8]. The majority of cc RCCs exhibit VHL mutations and/or silencing by methylation, which leads to enhanced HIF action and a hypoxic pattern of gene expression [2,3]. VHL also has a number of other important functions that may have tumour suppressor effects (for review, see [9]).

Many nonrenal cancers are also characterised by hypoxia, enhanced HIF levels and increased expression of hypoxically regulated genes which correlate both with tumour aggression and patient outcome [4]. Recently, comprehensive gene arrays have emphasised the dominant role of the HIF-VHL transcriptional system and the HIF peptidyl hydroxylases in the regulation of gene expression by hypoxia [7] and have characterised HIF-dependent and HIF-independent pathways of transcriptional regulation in VHL-deficient cells. However, other mechanisms of gene regulation by hypoxia include control of mRNA stability, regulation of mRNA translation, global influences on the transcriptional machinery and regulation mediated by microRNAs (miRNAs) [10].

miRNAs are noncoding RNA oligonucleotides that function as important regulators of gene expression. Differential levels of specific miRNAs have been observed in several tumour types when compared to normal tissue [11,12]. In addition, global reductions in miRNA expression are a feature of many cancers, miRNA gene copy number variation appears common in cancer, and overexpression of miRNAs can contribute to oncogenesis [13-15]. mRNA targets of these miRNAs include genes encoding proteins with roles in apoptosis, cell cycle and growth (for review, see [16]). Furthermore, certain tumour suppressors such as p53 can directly influence miRNA production [17], and gene polymorphisms of components of the miRNA biogenesis machinery have been associated with renal cell carcinoma susceptibility [18]. The precise mechanisms underlying inducible production of miRNAs, the role of tumour suppressors, such as VHL, in miRNA regulation and the effects of the HIF-VHL system on miRNA activity require further definition, as does the contribution of VHL-dependent alterations in miRNA abundance to the pathogenesis of renal cancers.

To determine the possible role of miRNAs in hypoxic gene regulation and to examine for hypoxically regulated miRNAs that may have relevance to tumour pathogenesis, we have surveyed changes in miRNA expression levels in response of breast cancer cells to hypoxia and characterised the hypoxic regulation of one specific human miRNA (miR-210) [19,20]. Given the link between hypoxia and miR-210 regulation and the recognition of particular alterations in miRNA expression in cancer, we hypothesised that levels of miR-210 expression in cancer would correlate with degree of hypoxia and tumour behaviour. To test this hypothesis, we have recently examined the expression of this hypoxically induced miRNA in human breast cancers and found a striking association with breast cancer prognosis [19,20]. Given the central role of VHL in hypoxic gene regulation and in renal cancer formation, we wished to examine the VHL-dependent regulation of miRNAs in renal cancer. miRNA dysregulation has been observed in renal cancer, and miR-210 has been identified as a hypoxia and VHL-regulated miRNA in renal cancer cells [21-23]. We wished to understand the mechanism underlying such alterations, examine the extent to which VHL-mediated alterations in miRNAs were HIF-dependent or independent and examine for the utility of miRNA alterations in renal cancer diagnosis.

## Methods

### Cell lines

The renal cancer cell line RCC4 stably transfected with either an empty vector or a vector encoding VHL was used in this study [24]. Cells were grown in Dulbecco's modified Eagle's medium supplemented with 10% fetal bovine serum (Invitrogen, Newcastle, NSW, Australia). All experiments were conducted in triplicate with independent cell cultures.

### Cell treatments

Treatment of cells with dimethyloxalylglycine (DMOG) (Biomol International, Plymouth Meeting, PA, USA) involved supplementing cell media with 1 mM DMOG diluted in phosphate-buffered saline (PBS) for 24 hours [25,26].

For HIF-1α and HIF-2α small interfering RNA (siRNA) treatments, RCC4-VHL cells were seeded at 30-50% confluency and grown for 24 hours. Cells were then transfected with 50 mM HIF-1α (sense 5'-CUGAUGACCAGCAACUUGAdTdT-3' and antisense 5'-UCAAGUUGCUGGUCAUCAGdTdT-3'; Dharmacon, Lafayette, CO, USA), HIF-2α siRNA (sense 5'-CAGCAUCUUUGAUAGCAGUdTdT-3' and antisense 5'-ACUGCUAUCAAAGAUGCUGdTdT-3'; Dharmacon) [19] and Accell negative control kit (Dharmacon) using Lipofectamine 2000 (Invitrogen). After 48 hours, RNA and protein were extracted.

For miR-210 overexpression studies, 20 nM miR-210 oligonucleotide duplex (sense 5'-CUGUGCGUGUGACAGCGGCUGA-3' and antisense 5'-AGCCGCUGUCACACGCACAGUU-3'; GenePharma, Shanghai, China) and 20 nM mimic negative control (sense 5'-UUCUCCGAACGUGUCACGUTT-3' and antisense 5'-ACGUGACACGUUCGGAGAATT-3'; GenePharma) were transfected into RCC4 + VHL cells seeded at 30-50% confluency. RNA was harvested after 48 hours.

For miR-210 repression studies, 20 nM miR-210 siRNA (5'-UCAGCCGCUGUCACACGCACAG-3'; GenePharma) and 20 nM miRNA inhibitor negative control (5'-UCUACUCUUUCUAGGUUGUGA-3'; GenePharma) were transfected into RCC4-VHL cells seeded at 30-50% confluency. RNA was harvested after 48 hours.

### Microarray

The miRNA microarrays consisted of 1488 antisense miRNA oligonucleotide probes (miRCURY LNA microRNA probe set, catalog no. 208010 V8.1; Exiqon, Vadbaek, Denmark) printed in duplicate onto epoxide-coated microarray slides (Corning Life Sciences, Acton, MA, USA). For detection on the array, 5 μg of total RNA was labelled by the ligation of a fluorescently modified RNA dimer [27]. Two sample (dual colour) competitive hybridizations were performed using Cy3- and Cy5-labelled sample pairs.

Hybridisation was performed for 16 hours at 56°C under LifterSlips (Erie Scientific, Portsmouth, NH, USA) in 1× Exiqon hybridization buffer (catalog no. 208020) (Exiqon, Vadbaek, Denmark) in a total volume of 25 μL.

Slides were placed in Corning hybridization chambers and protected from light for the 16-hour incubation. Slides were washed using dilutions of the Exiqon Wash Buffer kit (catalog no. 208021) as recommended by the manufacturer. Slides were scanned at 10-μm resolution with a Genepix 4000B Scanner (Molecular Devices, Union City, CA, USA).

Median spot pixel intensity values in scanned images were extracted using the Spot v3 plugin (CSIRO, Clayton South, VIC, Australia) for the statistical environment R. After subtraction of background intensities and global loess normalisation, mean intensities were log_2 _transformed and ratios (Cy5/Cy3) were obtained. Differentially expressed miRNAs were determined using linear models and empirical Bayesian moderation of standard errors (LIMMA R package, WEHI, Melbourne, VIC, Australia; [28]).

### Immunoblotting

Whole cell extracts were resolved by standard polyacrylamide gel electrophoresis and electroblotted onto polyvinylidene difluoride membrane (Millipore, Bedford, MA, USA). Primary antibodies used were mouse monoclonal anti-HIF-1α (610958; BD Transduction Laboratories, San Diego, CA, USA) and rabbit polyclonal anti-HIF-2α (NB100-122; Novus Biologicals, Littleton, CO, USA). Horseradish peroxidase-conjugated secondary antibodies goat anti-rabbit IgG and donkey anti-mouse IgG (Immunopure; Thermo Scientific, Rockford, IL, USA) were used in conjunction with the ECL system (SuperSignal West Pico; Pierce, Rockford, IL, USA) to visualise bands using an ImageQuant LAS 4000 system (GE Healthcare Life Sciences, Uppsala, Sweden).

### Patient samples

Thirty-one patients with cc RCC treated in Adelaide, South Australia, Australia, between 1997 and 2006 were studied. Samples were obtained from the Flinders Medical Centre/Repatriation General Hospital Tissue Bank facility. Ethical approval for analysis of samples was obtained from the Flinders Clinical Research Ethics Committee (FCREC). All patients gave informed, signed consent. The clinical details of the patients are summarised in Table 1.

**Table 1:** Summary of the clinical characteristics of 31 patients with cc RCC.

Clinical Features	Number of patients
Sex	
Female	6
Male	25
Age (yr)	
Median	67
Range	31-88
Grade	
1	3
2	16
3	10
4	2
TMN Stage	
pT1	5
pT2	6
pT3a	8
pT3b	2
pT4	1
Unknown	9
Size (mm)	
21-50	18
51-100	7
101-150	4
151-200	0
201-250	1
Unknown	1

Pathological details of cc RCC tumours according to Fuhrman's grading system and the International Union Against Cancer (UICC) TNM classification. The size of tumours (in mm) and the age and gender of the 31 patients is also shown.

### Quantitative real-time PCR for miRNAs, CAIX and ISCU1/2

miRNA expression was assessed by relative quantitation real-time PCR (qPCR) using TaqMan microRNA assays (Applied Biosystems, Foster City, CA, USA).RNA was extracted from cells and tissue using TRIzol reagent (Invitrogen) according to the manufacturer's instructions. RNA quantity and quality was determined using a Nanodrop-8000 spectrophotometer (Nanodrop Technologies, Wilmington, DE, USA). cDNA was synthesised from 5 ng of total RNA using TaqMan miRNA-specific primers and the TaqMan microRNA reverse transcription kit (Applied Biosystems). qPCR was performed using the Corbett Rotor-gene 2000. Each PCR was performed in triplicate and contained 1 μl of reverse transcription product, 1× TaqMan Universal PCR Master Mix No AmpErase UNG and 0.5 μl of primer and hydrolysis probe mix of the TaqMan microRNA assay (assay IDs: miR-210: 000512, miR-155: 000479, miR-21: 000397, miR-31: 001100, miR-20a: 000580, miR-18a: 002422, miR-17: 000393, let-7i: 002221, miR-193b: 001010; Applied Biosystems). The 10-μl reactions were incubated at 95°C for 10 minutes, followed by 40 cycles of 95°C for 15 seconds and 60°C for 60 seconds. Results were normalised to the expression of the small nuclear RNA gene RNU6B (assay ID: 001093; Applied Biosystems) for cell samples and an average of the small nucleolar RNAs RNU43 and RNU48 (assay IDs: 001095 and 001006, respectively; Applied Biosystems) expression levels for tissue samples, as no significant variation of expression was found between tumour and normal tissue. Data were generated and analysed using Corbett Rotorgene software (version 5.0.61) (Corbett Research, Sydney, NSW, Australia) and the Relative Expression Software Tool (REST) program (Corbett Research, Sydney, NSW, Australia) [29].

For mRNA expression studies, 1 μg of RNA was reverse transcribed following DNase treatment (New England Biolabs, Beverly, MA, USA) using M-MLV Reverse Transcriptase RNase H minus, Point mutant (Promega, Madison, WI, USA) and Oligo dT(15) primers. Carbonic anhydrase IX (CAIX) mRNA expression was assessed by qPCR according to the SYBR Green protocol (Applied Biosystems). The following primers were used to amplify CAIX (each at 0.25 μM per reaction): forward 5'-CCTCTCCCGGAACTGAGCCTAT-3' and reverse 5'-TGTTCTGAGCCTGGGTGATCTG-3' [30]. Iron-sulfur cluster assembly (ISCU1/2) mRNA expression was assessed by qPCR using the ISCU1/2 TaqMan gene expression kit (assay ID: Hs00384510_m1; Applied Biosystems) according to the manufacturer's instructions, and the human β-actin gene was used as a reference using the following primers (each at 0.25 μM per reaction): forward 5'-TTGCCGACAGGATGCAGAAG-3' and reverse 5'-GCCGATCCACACGGAGTACT-3'.

### VHL sequencing

Mutation analysis of the promoter region, three exons and associated splice junctions of the VHL gene were performed by PCR amplification and cycle sequencing. Genomic DNA was extracted from tumour samples using the DNeasy kit, spin-column protocol (Qiagen, Hilden, Germany) according to the manufacturer's instructions. DNA was PCR amplified in 50-μl reactions using Ampli*Taq*Gold (Roche, Branchburg, NJ, USA)according to the manufacturer's instructions and the following conditions and primers. Promoter and Exon 1: Primers forward 5'-TAGCCTCGCCTCCGTTACA-3' and reverse 5'-GCTTCAGACCGTGCTATCG-3', Exon 2: Primers forward 5'-TGATCTCCTGACCTCATGAT-3' and reverse 5'- GACACCATAACACCTTTAAC-3' and Exon 3: Primers forward 5'-TACTGAGACCCTAGTCTG-3' and reverse 5'-GGAAGGAACCAGTCCTG-3'. PCR primers for exons 1 and 3 were tagged with universal M13 primers. PCR products were visualised on agarose gel and purified using ExoSAP-IT (GE Life Sciences) according to the manufacturer's instructions. Cycle sequencing was performed using universal M13 forward and reverse primers for exons 1 and 3 and the PCR primers for exon 2 and standard BigDye (Applied Biosystems) chemistry. Sequencing products were purified using Agencourt CleanSEQ Dye Terminator Removal (Beckman Coulter, Beverley, MA, USA) according to the manufacturer's instructions and analysed on an Applied Biosystems 3730 analyser.

Sequence traces were compared to the National Center for Biotechnology Information (NCBI) reference sequence NM_000551.2 using Mutation Surveyor (SoftGenetics, State College, PA, USA). Splice site variants were analysed using the Berkeley Drosophila Genome Project (BDGP) http://www.fruitfly.org/about/index.html, NetGene2 http://www.cbs.dtu.dk/services/NetGene2/ and GeneSplicer http://www.cbcb.umd.edu/software/GeneSplicer/gene_spl.shtml splice site predictors. Human Genome Variation Society (HGVS) nomenclature was used for variant identification [31].

### VHL promoter methylation analysis

Bisulphite treatment followed by methylation-specific PCR (MSP) was used to analyse the methylation status of the VHL promoter [32]. Genomic DNA was subjected to bisulphite treatment using an EpiTect Bisulfite kit (Qiagen, Hilden, Germany) according to the manufacturer's instructions. The following primers were used for amplifying the VHL promoter: Unmethylated-specific (expected size 165 bp) forward 5'-GTTGGAGGATTTTTTTGTGTATGT-3', reverse 5'-CCCAAACCAAACACCACAAA-3', and methylated-specific (expected size 158 bp) forward 5'-TGGAGGATTTTTTTGCGTACGC-3', reverse 5'-GAACCGAACGCCGCGAA-3' [33]. The PCR mixture contained 1× PCR buffer (New England Biolabs), deoxynucleotide triphosphates (each at 2.5 mM), primers (each at 0.8 mM), 50 ng modified DNA and 2.5 U of Taq DNA polymerase (New England Biolabs) added after a 5-minute at 95°C hot start, in a total reaction volume of 50 μl. Reactions were carried out on a GeneAmp PCR system 9700 thermocycler (Applied Biosystems) for 35 cycles (30 seconds at 95°C, 30 seconds at 47°C and 30 seconds at 72°C), followed by a final extension of 4 minutes at 72°C. Each PCR was loaded (20 μl) onto a 3% agarose gel containing ethidium bromide and visualised under UV light.

### Statistical analysis

All statistical analysis was done using PASW Statistics 17 (Somers, NY, USA). The Mann-Whitney *U *test was used to assess the difference between miRNA expression in RCC4 ± VHL cells, treated/untreated cells and tumour/normal adjacent tissue. Spearman's ρ correlation coefficient was used to assess correlations between miRNA expression and ISCU1/2 and CAIX expression and for the correlation of miR-210, miR-155, miR-21, miR-18a, let-7i, ISCU1/2 and CAIX expression with survival, tumour size, grade and tumour node metastasis (TNM) stage. Kaplan-Meier log-rank (Mantel-Cox) analysis was undertaken to assess overall survival, miR-210 expression and TNM stage. Results were considered statistically significant at *P *≤ 0.05.

## Results and Discussion

### VHL regulation of miRNA expression in RCC4 cells

**Figure 1:**
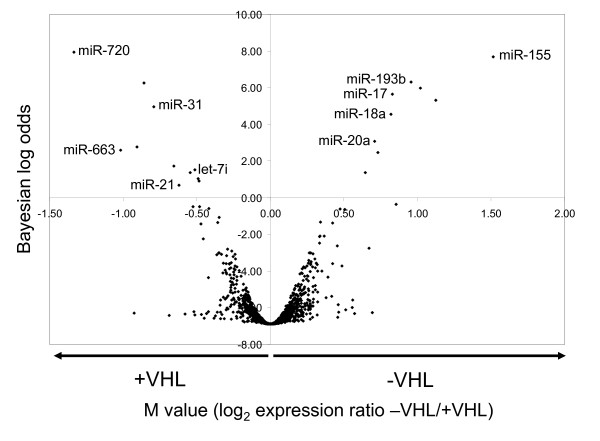
**MicroRNA (miRNA) expression in RCC4 cells assayed by microarray**. The *x*-axis represents the differential expression (M value = log_2_) in renal cancer cells with and without von Hippel-Lindau (VHL) disease. The *y*-axis represents the (Bayesian) probability that miRNA is differentially expressed. The top five up- and downregulated differentially expressed human miRNAs are labelled. The array was performed using three independent samples of each cell type.

To test the hypothesis that there are specific alterations in miRNA expression in VHL-dependent renal cancer, we studied parallel cultures of renal carcinoma cells with mutated VHL (RCC4-VHL) and reintroduced wild-type VHL (RCC4 + VHL). We analysed these cells for expression of both HIF-1α and HIF-2α and, in keeping with previous studies [24], confirmed high levels of HIF in cells lacking functional VHL (Additional file 1). We undertook a microarray analysis of miRNA expression in RCC4 cells and compared expression in cells with and without functional VHL (Figure 1).

**Figure 2:**
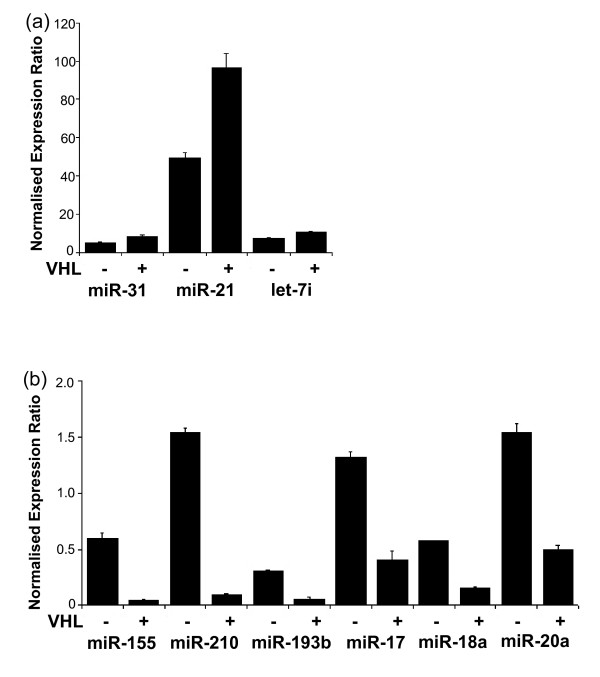
**VHL regulation of miRNA expression in RCC4 cells**. Expression of VHL-regulated miRNAs was validated by relative quantitation real-time PCR (qPCR). **(a) **VHL-dependent downregulation of miR-31, miR-21 and let-7i in RCC4-VHL cells (*P *= 0.05). **(b) **VHL-dependent upregulation of miR-155, miR-210, miR-193b, miR-17, miR-18a and miR-20a in RCC4-VHL (*P *= 0.05). The expression of the miRNAs was normalised to RNU6B expression. The mean ± SEM of three independent cell cultures is shown, and each sample was assayed in triplicate.

Whilst the majority of miRNAs showed no substantial difference in expression, some showed very significant VHL-dependent differences. miRNAs with a positive Bayesian log odds value were considered to be differentially expressed (Figure 1). Furthermore, we sought to confirm these findings by measuring the expression of the top five up- and downregulated human miRNAs in RCC4-VHL by qPCR in independent experiments, in addition to miR-210, which we had previously shown to be regulated by the HIF-VHL system [34]. Of the selected downregulated miRNAs, miR-21, miR-31 and let-7i were found to be slightly repressed 1.9-, 1.7- and 1.4-fold, respectively; *P *= 0.05 (RCC4-VHL compared to RCC4 + VHL) (Figure 2a). No TaqMan assay is currently available to validate miR-663 expression by qPCR, and the downregulation of miR-720 was not detectable in our assays. All of the selected upregulated miRNAs, miR-155, miR-193b, miR-18a, miR-20a and miR-17 (also known as miR-17-5p), were found to be highly expressed in cells without functional VHL (13.0-, 5.2-, 3.8-, 3.1- and 2.3-fold, respectively; *P *= 0.05) (RCC4-VHL compared to RCC4 + VHL) (Figure 2b). Consistent with other reports [35], the amplitude of differential expression was higher than had been assessed with the microarray platform. Furthermore, the differential expression of miR-210 was confirmed (16-fold; *P *= 0.05) (RCC4-VHL compared to RCC4 + VHL) (Figure 2b). The results were controlled and normalised by reference to expression of RNU6B, whose levels we have previously shown to be unaffected by VHL status and hypoxia [34].

**Figure 3:**
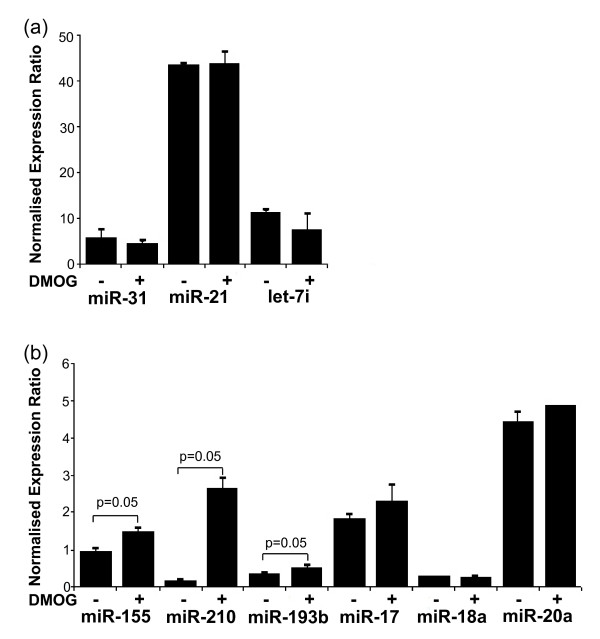
**miRNA expression in RCC4 + VHL cells following dimethyloxalylglycine (DMOG) treatment**. Expression of VHL-regulated miRNAs in RCC4 + VHL cells in response to DMOG treatment was assessed by qPCR. **(a) **Expression of miR-31, miR-21 and let-7i is unchanged in response to DMOG treatment. **(b) **Expression of miR-210, miR-155 and miR-193b is increased upon treatment with DMOG in RCC4 + VHL cells (*P *= 0.05), but the expression of miR-17, miR-18a and miR-20a is unchanged. The expression of the miRNAs was normalised to RNU6B. The mean ± SEM of three independent cell cultures is shown, and each sample was assayed in triplicate.

Although the microarray analysis may not have detected the expression of all VHL-regulated miRNAs, the subsequent qPCR validation confirms the existence of substantial VHL-dependent regulation of multiple specific miRNAs in cell culture. The VHL-dependent miRNA expression pattern we observed might be mediated by enhanced levels of HIF-α proteins or alternatively might be due to other effects of VHL. HIF-independent functions of VHL have been described in cellular senescence, microtubule stability and matrix formation [9]. To examine the extent to which this pattern of VHL-dependent miRNA dysregulation was mediated by the HIF system, we examined the effects of the prolyl hydroxylase inhibitor DMOG, which activates HIF and a HIF-dependent pattern of gene expression [7,26]. We confirmed this induction of HIF-1α and HIF-2α by DMOG in the RCC4 + VHL cells (Additional file 1) and also confirmed the induction of the HIF target, CAIX, by DMOG in the RCC4 + VHL cells (Additional file 2). Of the five miRNAs confirmed to be upregulated in RCC4-VHL cells, only miR-155 and miR-193b were found to be slightly upregulated in response to DMOG treatment in RCC4 + VHL (1.6- and 1.5-fold, respectively; *P *= 0.05) (Figure 3b), although the amplitude of induction was less than that seen between cells differing in VHL status. Figure 3a shows that the three miRNAs (miR-31, miR-21 and let-7i) confirmed to be downregulated in the RCC4-VHL cell line are not downregulated in response to DMOG treatment of the RCC4 + VHL cell line. As expected, the expression of miR-210 also increased with DMOG treatment (15-fold; *P *= 0.05) (Figure 3b).

**Figure 4:**
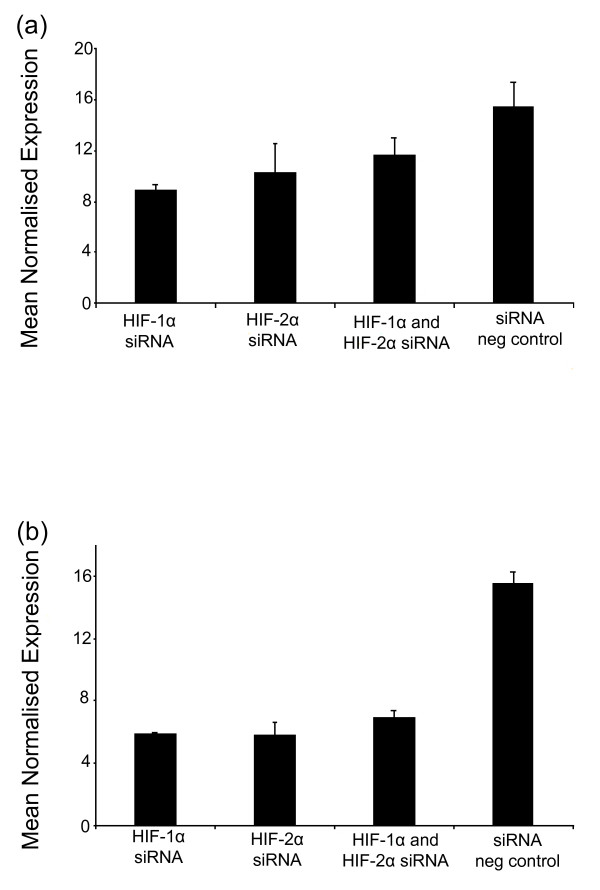
**miRNA expression in RCC4-VHL cells following hypoxia-inducible factor (HIF) small interfering RNA (siRNA) treatment**. Expression of VHL-regulated miRNAs in RCC4-VHL cells in response to HIF-1α and HIF-2α siRNA treatment was assessed by qPCR. **(a) **Expression of miR-210 is decreased in response to HIF siRNA treatment in RCC4-VHL cells (*P *= 0.05). **(b) **Expression of miR-155 is also decreased upon treatment with HIF siRNA in RCC4-VHL cells (*P *= 0.05). The expression of the miRNAs was normalised to RNU6B. The mean ± SEM of three independent cell cultures is shown, and each sample was assayed in triplicate.

To further explore whether the miRNAs induced in the absence of VHL are indeed regulated by HIF, we transfected the RCC4-VHL cell line with HIF-1α and HIF-2α siRNA oligonucleotides. Figure 4 shows that following treatment to suppress HIF expression, the levels of miR-210 and miR-155 were decreased in the treated lines compared to those treated with a negative transfection control. HIF-1α siRNA treatment decreased miR-210 and miR-155 levels 1.75- and 2.8-fold, respectively, and HIF-2α siRNA treatment decreased miR-210 and miR-155 levels 1.6- and 2.8-fold, respectively (*P *= 0.05), in keeping with the results of HIF hydroxylase inhibition and consistent with a role for HIF in their control. However, miR-193b did not show any change between treated and control cells, which may indicate that miR-193b regulation is only partially mediated by HIF. Furthermore the lesser effect on miRNA regulation seen with HIF repression compared to that seen with DMOG treatment may represent insufficient duration of HIF repression or incomplete transfection efficiency in these cells. Taken together, these results suggest that the induction of several miRNAs in cells lacking VHL is mediated in large part via HIF induction (miR-210 and miR-155), whilst the dysregulation of other miRNAs (miR-31/miR-21/miR-18a/miR-17/let-7i/miR-20a) is HIF-independent and mediated by other actions of VHL.

### Analysis of VHL-dependent miRNA expression in renal tumours

Given these interesting observations in one particular renal cancer cell line, we examined whether this signature of VHL-dependent miRNA dysregulation occurs in renal tumours. This would provide information about the extent to which these specific VHL-dependent miRNA alterations are more widely observed in renal cancer, with important implications for understanding renal oncogenesis and developing diagnostic and prognostic markers in renal cancer.

**Figure 5:**
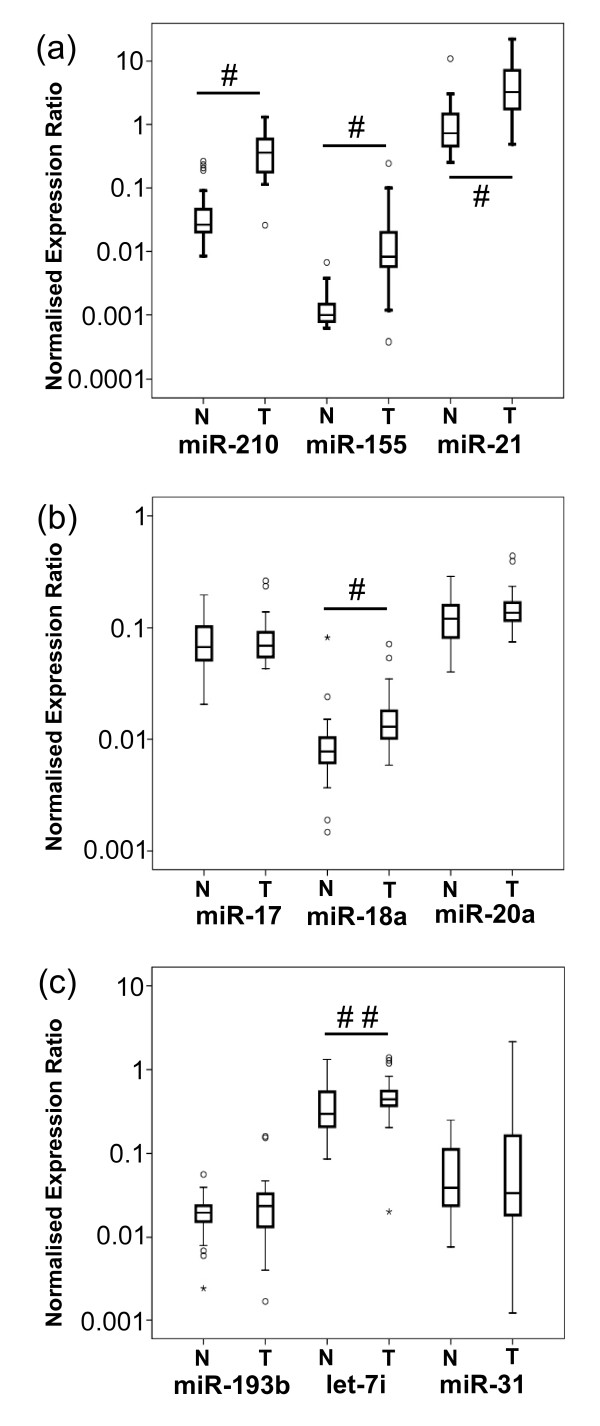
**Expression of VHL-regulated miRNAs in clear cell renal cell carcinoma (cc RCC) tissue compared to normal adjacent tissue**. Measurements of miRNA expression were determined by qPCR and normalised to an average of RNU43 and RNU48 expression. qPCR for each sample was performed in triplicate. **(a) **miR-210, miR-155 and miR-21 were all highly upregulated in tumour tissue compared to normal kidney tissue (*P *≤ 0.0001). **(b) **Expression of three members of the miR-17-92 cluster: miR-17, miR-18a and miR20a were examined, and miR-18a was found to be significantly upregulated in tumour tissue (*P *≤ 0.0001). **(c) **No significant difference in expression was observed for miR-193b and miR-31; however, let-7i displayed significant upregulation in tumour tissue (*P *≤ 0.043). #*P *≤ 0.0001; ##*P *≤ 0.05;°outlier (values between 1.5 and 3 interquartile ranges (IQRs) from the end of box); *extreme outlier (values more than 3 IQRs from the end of box).

To address the hypothesis that VHL-regulated miRNAs are also dysregulated in renal tumours, we examined the nine miRNAs found to be differentially expressed between RCC4-VHL and RCC4 + VHL cell lines. The expression of several of these miRNAs was found to be upregulated in 31 cc RCC tumours compared to adjacent normal tissue (Figure 5). The expression of each miRNA was normalised to an average of RNU43 and RNU48 expression, both of which were found not to be differentially expressed between tumour and normal tissue (0.96-fold (*P *= 0.906) and 1.02-fold (*P *= 0.746) expression in tumour relative to adjacent normal tissue, respectively). Figure 5a shows the expression of miR-210, miR-155 and miR-21, which are significantly upregulated in tumour tissue compared to normal adjacent tissue (8-, 16- and 4-fold, respectively; *P *≤ 0.0001). The expression of three members of the miR-17-92 cluster in tumour tissue was also examined. miR-18a was upregulated in clear cell tumour tissue compared to adjacent normal tissue (1.6-fold; *P *≤ 0.0001), however, while miR-20a also appeared to be slightly upregulated (1.3-fold), this was not statistically significant (*P *= 0.067). The expression of miR-17 was not found to be significantly different in tumour compared to normal adjacent tissue (Figure 5b). The miRNAs miR-193b and miR-31 were not significantly different between tumour and adjacent normal tissue; however, let-7i was slightly upregulated 1.2-fold in tumour tissue (*P *= 0.043) (Figure 5c). Of interest are four normal tissue samples in Figure 5a which are outliers and display miR-210 levels as high as the mean tumour expression level. It is possible that the normal kidney tissue taken from these patients was diseased or hypoxic as a consequence of the adjacent tumour, leading to abnormally high miR-210 expression levels.

These results show that some of the VHL-regulated miRNAs, such as miR-210 and miR-155 in cultured cells, are highly differentially expressed between renal cancer tissue and normal tissue. However, other miRNAs such as miR-31 and miR-193b do not show such regulation and indeed, somewhat surprisingly, miR-21 and let-7i, which were decreased in cells lacking VHL, were overexpressed in renal cancer when compared to normal adjacent renal tissue. This suggests that despite VHL regulation at the cellular level, tumour growth and progression select for enhanced miR-21 and let-7i expression in tumour cells or other cells within the renal cancers. The decrease in expression in RCC4-VHL cells may be an artefact of cell culture resulting from different selection pressures in culture compared to cc RCC tumour tissue. Also, there are many mechanisms that may lead to overexpression of miR-21 in renal cancer, and these may not be related to VHL function. The RCC4 cell line used in this study may not be representative of the situation in all renal cancers, and in future studies it would be beneficial to validate these findings in additional kidney cancer cell lines.

miR-21 has been identified as having oncogenic properties and is overexpressed in the majority of cancer types examined [36]. A host of tumour suppressor genes have now been validated as targets of miR-21 (for review, see [36]), and recently the suggestion has been made, on the basis of bioinformatics analysis, that VHL may be a target of miR-21 [22,37], but this awaits experimental validation. Our finding that miR-21 is upregulated in cc RCC tumours is in keeping with two recent studies profiling miRNA expression in renal cancer that have also identified miR-21 as upregulated in cc RCC tumour tissue compared to normal kidney tissue, although the increase in expression observed in our study (fourfold) was higher than previously reported (2.5- and 1.2-fold) [21,22].

The overexpression of miR-210 in cc RCC has also recently been reported [21-23]. miR-210 is also overexpressed in many other tumour types [16], most notably breast cancer, in which it was found to be a prognostic marker [19]. miR-155 is also overexpressed in many cancers [16] and has been shown to inhibit apoptosis by targeting tumour protein p53 inducible nuclear protein 1 (TP53INP1) [38]. In addition, O'Connell and others have found that inositol phosphatase SHIP1 is a target of miR-155 [39]. Our results are in accord with those of other studies which have found increased miR-155 expression levels in cc RCC tissue, although the reported fold increases (3.2- and 6.4-fold) [21,23] were less than what was found in this series (15-fold).

The differential expression of some members of the miR-17-92 cluster in cc RCC is particularly interesting, as overexpression of this miRNA cluster has been implicated in a wide range of cancers and has been shown to act as an oncogene [13-15]. miR-17-92 has been shown to play an important role in tumour cell proliferation and apoptosis [16,40], to negatively regulate HIF-1α expression [41] and appears to have a critical function in vascular endothelial growth factor-induced angiogenesis [42]. Under normal physiological conditions, miR-17-92 is involved in the regulation of MYC-induced cell proliferation by inhibiting E2F1 expression; however, when miR-17-92 is overexpressed in many cancers, it can act with MYC to synergistically contribute to aggressive cancer development [14,43-45]. The overexpression of three members of the miR-17-92 cluster, miR-17, miR-20a and miR-18a, has previously been reported for cc RCC [21,37]. Our finding that these three miRNAs are upregulated in the RCC4-VHL cell line is in accord with findings in these studies; however, we were unable to confirm the upregulation of miR-17 in tumour tissue or the statistically significant upregulation of miR-20a. This discrepancy may be due to small sample size, differences in procedure or potential interference of other cell types in the tumour samples.

Eleven members of the let-7 family have been identified in the human genome. The family was one of the earliest tumour suppressor miRNAs identified in cancer, and some members of the let-7 family can also have oncogenic roles [46]. The increased expression of one member of the let-7 family, let-7i, has been reported in breast cancer [47] and head and neck squamous cell carcinoma [48], and decreased expression is associated with chemotherapy resistance and shorter progression-free survival in late-stage ovarian cancer patients [49]. To the best of our knowledge, this is the first report of a change in let-7i expression in renal cancer.

### The hypoxic and VHL status of the tumour tissue

**Figure 6:**
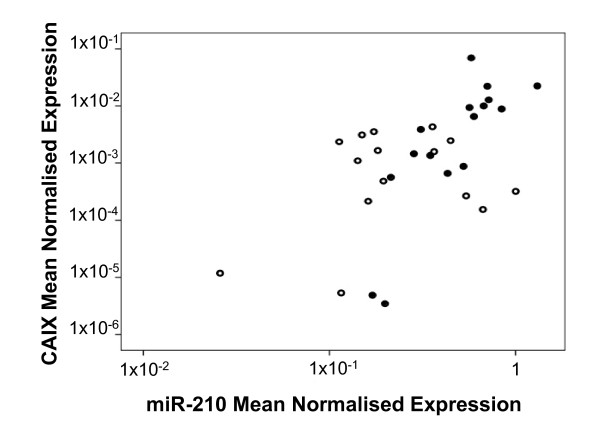
**Correlation between carbonic anhydrase IX (CAIX) and miR-210 expression**. Measurements of CAIX mRNA and miR-210 expression were determined by qPCR and normalised to β-actin mRNA expression and an average of RNU43 and RNU48 expression, respectively. qPCR for each sample was performed in triplicate. A significant positive correlation is observed between the mean normalised expression of CAIX and miR-210 (*r *= 0.527, *P *= 0.002). Data are displayed on log_10 _axes. Shaded circles indicate tumours which have VHL mutation and/or VHL promoter methylation, and open circles represent tumours for which no VHL defect has been identified.

As some of the miRNAs display HIF-dependent regulation (Figure 3b and Figure 4), we sought to examine HIF-dependent mRNA expression in the 31 tumour samples. CAIX is a transcriptional target of HIF-1. Its expression has previously been shown to be an intrinsic marker of hypoxia/HIF activation [50] and has been reported to be a predictor of outcome in renal cancer [51]. Thus, the expression level of CAIX in the cc RCC tumour tissue was determined to infer the presence of hypoxia and/or the likely activation of HIF. Consistent with this, an undetectable level of expression was observed in the normal matched adjacent tissue. Furthermore, a striking positive correlation was found between CAIX and miR-210 expression (*r *= 0.527, *P *= 0.002), as shown in Figure 6. No correlation was observed between CAIX levels and other VHL-regulated miRNAs, with the exception of miR-155 which showed a weak correlation (*r *= 0.318, *P *= 0.082). This is consistent with the regulation of miR-155 by VHL through HIF-1α. These results provide additional support for the concept that several of the VHL-dependent miRNAs are at least partially regulated via HIF whilst others are regulated by HIF-independent mechanisms.

**Figure 7:**
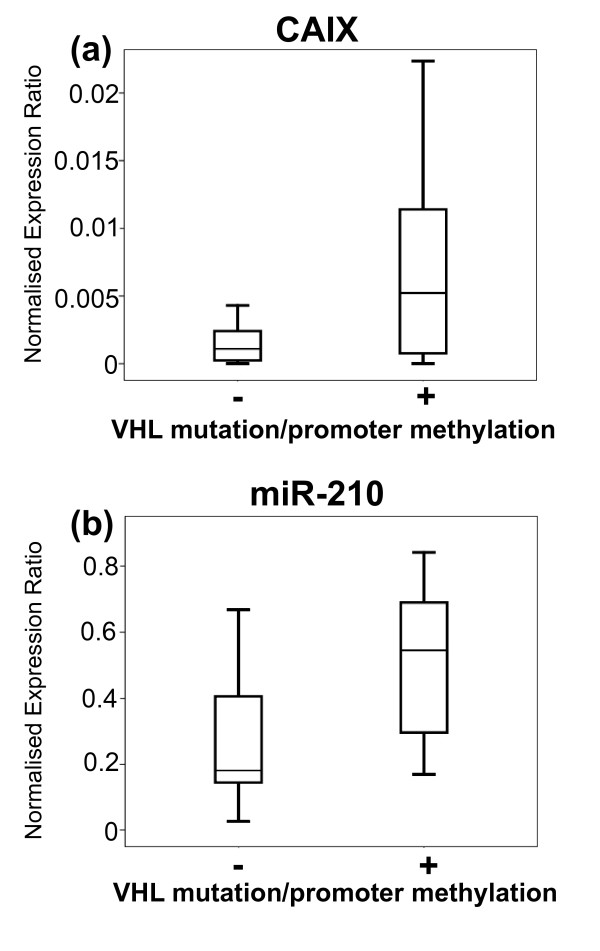
**CAIX and miR-210 expression in tumours with and without VHL mutation and VHL promoter methylation**. Measurements of CAIX mRNA and miR-210 expression were determined by qPCR and normalised to β-actin mRNA expression and an average of RNU43 and RNU48 expression, respectively. qPCR for each sample was performed in triplicate. Expression of CAIX and miR-210 was found to be increased in tumours with VHL mutation or promoter methylation compared to tumours in which neither was identified (*P *= 0.045 and 0.011, respectively).

We also analysed miRNA and CAIX expression by whether the tumours had identifiable VHL defects. The tumours were analysed for VHL mutations and VHL promoter methylation status (Additional file 3). It has been found that biallelic VHL mutations are found in 50-75% of sporadic cc RCC tumours [2,3]. In keeping with other series, VHL mutations were found in 42% of clear cell tumours [33], and the VHL promoter was found to be methylated in 10% of clear cell tumours [52,53]. We hypothesised that CAIX and HIF-regulated miRNAs would be indicative of the VHL status of the tumour, and indeed a significant difference between the two groups was observed for CAIX and miR-210 expression. CAIX and miR-210 expression were found to be significantly increased in tumours with identifiable VHL mutations, or promoter methylation, compared to tumours in which we did not identify a mechanism for suppression of VHL function (*P *= 0.045 and 0.011; Figures 7a and 7b, respectively).

However, a substantial proportion of the tumours without identified VHL inactivation still display high expression of CAIX and miR-210. Whilst it is possible that our mutational and methylation analysis did not identify all of the causes of VHL inactivation present in these tumours, it indicates that they may have other genetic mutations that influence VHL or HIF expression or function. These mechanisms potentially include disruptions in SETD2 (histone H3K36 methyltransferase) or JARID1C (histone H3K4 demethylase), which have both been recently identified as having truncating mutations in approximately 3% of cc RCC tumours [54]. Other potential mechanisms of VHL suppression might be mediated via miRNAs, as has been reported recently for miR-92 in chronic lymphocytic leukaemia [55]. The mechanisms leading to cc RCC tumour initiation and/or activation of hypoxic pathways in tumours without VHL mutation or VHL promoter methylation are not well understood, and further work is needed.

### Correlation of miRNA level with clinical features

**Figure 8:**
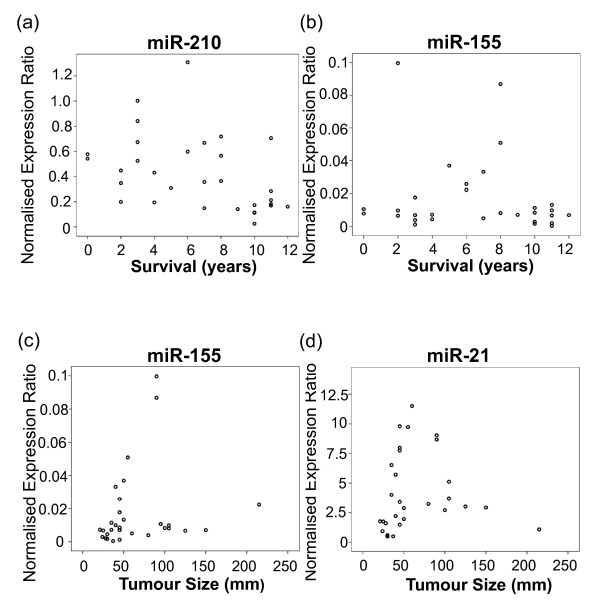
**Correlation of miRNA expression with clinical features**. Measurements of miRNA expression were determined by qPCR and normalised to an average of RNU43 and RNU48 expression. qPCR for each sample was performed in triplicate. **(a) **A significant correlation was found between miR-210 expression and survival (years) (*r *= -0.481, *P *= 0.006). **(b) **No significant correlation was found between miR-155 expression and survival (years). Significant correlations were found between **(c) **miR-155 and **(d) **miR-21 expression and tumour size (mm) (*r *= 0.408, *P *= 0.028 and *r *= 0.434, *P *= 0.019, respectively).

Overall the 5-year survival in the 31 cc RCC patients was 71%. A significant inverse correlation was found between miR-210 expression in cc RCC tumours and survival (years) (*r *= -0.481, *P *= 0.006) (Figure 8a). Weaker inverse correlations were also found between miR-21, let-7i and miR-18a expression in cc RCC tumour tissue and survival (years) (*r *= -0.308, *P *= 0.09; *r *= -0.339, *P *= 0.062; and *r *= -0.300, *P *= 0.101, respectively); however, the correlations did not achieve statistical significance. No correlation was found between miR-155 expression and survival (Figure 8b). The expected correlations between tumour size, grade, TNM stage and survival (years) were found (*r *= -0.572, *P *= 0.001; *r *= -0.354, *P *= 0.055; and *r *= -0.46, *P *= 0.031, respectively).

A significant correlation was also found between miR-155 and miR-21 expression in cc RCC tumours and tumour size (mm) (*r *= 0.408, *P *= 0.028; *r *= 0.434, *P = *0.019, respectively) shown in Figures 8c and 8d. Correlations were also observed for miR-210 and let-7i expression and tumour size (mm); however, these correlations did not achieve statistical significance (*r *= 0.356, *P *= 0.058; and *r *= 0.363, *P *= 0.053, respectively). No correlations were found between miRNA expression in cc RCC tumours and tumour grade or TNM stage.

**Figure 9:**
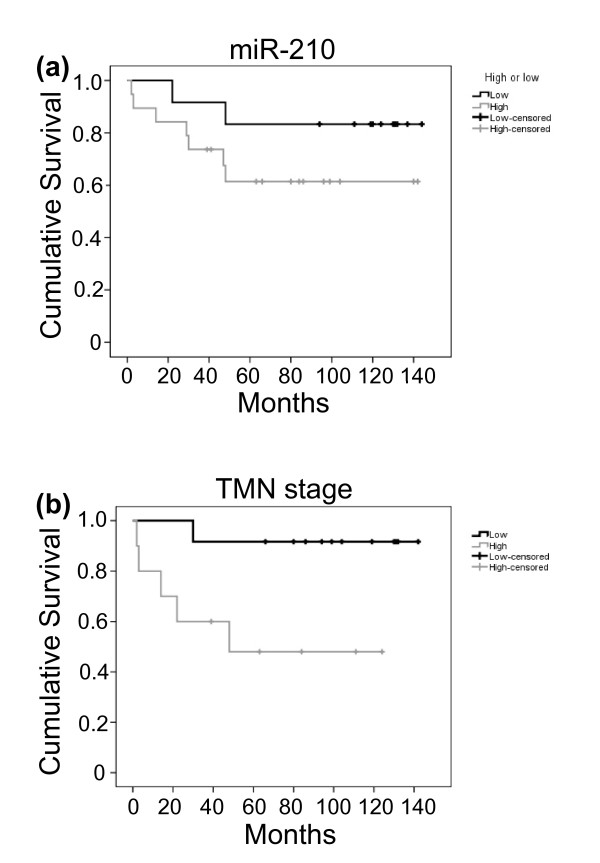
**Overall survival is associated with miR-210 expression and tumour node metastasis (TNM) tumour stage**. **(a) **Kaplan-Meier plot shows the overall survival of renal cancer patients stratified by high and low miR-210 expression levels. High expression is defined as above the maximum normal tissue expression value (*P *= 0.189; log rank). **(b) **Kaplan-Meier plot showing the overall survival of renal cancer patients stratified by low (Stages 1 and 2) and high (Stages 3 and 4) TNM stage cc RCC tumours (*P *= 0.022; log rank). The black line indicates low miR-210 expression or tumour stage, and the grey line indicates high miR-210 expression or tumour stage.

These results indicate the ability of levels of specific miRNAs to correlate with tumour features and patient outcome. The ability of levels of miR-210 to provide prognostic information has also been seen in breast cancer [19,20] and head and neck cancers [56]. Figures 9a and 9b show Kaplan-Meier plots that represent survival (months) stratified by high/low miR-210 expression and high/low TNM stage. An association was observed between both high miR-210 expression and high TNM stage with decreased survival (log-rank 0.189 and 0.022, respectively); however, the number of samples analysed in this study was too small for the miR-210 association to reach statistical significance. It should be noted that these are preliminary results and that additional confirmation of this result will now be required in other larger sets of renal cancers. In addition, it should be noted that in some renal tumours, HIF-2α expression may dominate and therefore miR-210 may not provide general prognostic information for all cc RCC tumours.

### Expression of ISCU1/2 in cc RCC tumours

The relationship between miR-210 levels and patient outcome suggests that miR-210 levels in cancer are not simply a marker of hypoxia but have a fundamental influence on tumour behaviour. To understand the mechanisms for the effects of miR-210 levels on patient outcome, we wished to study influences on potential mRNA targets. Computer-based algorithms are capable of predicting a large number of potential mRNA targets of particular miRNAs, some of which have been validated experimentally. We have defined a large number of potential miR-210 targets using such methodology, and during the course of this study Chan and colleagues [57] and subsequently others [58-60] were able to experimentally validate the regulation of one such predicted target, ISCU1/2.

The iron cluster assembly proteins, ISCU1 and ISCU2, are involved in the biogenesis of [4Fe-4S] and [2Fe-2S] iron-sulfur clusters, which are implicated in a wide range of biological processes, including electron transport and mitochondrial oxidation-reduction reactions [61]. The ISCU protein exists as two splice isoforms in mammalian cells which share both structural and functional similarities: ISCU1 is located in the cytosol, whereas ISCU2 is located in the mitochondria [62]. It has been hypothesised that miR-210 represses the expression of ISCU1/2 during hypoxic conditions, thus disrupting mitochondrial function and causing downstream metabolic changes within the cell [57-60].

We sought to examine whether the elevation of miR-210 levels in cc RCC was associated with reduced expression of ISCU1/2 mRNA. The expression of ISCU1/2 was analysed in the RCC4 ± VHL cell lines, and it was found that the expression of ISCU1/2 was significantly reduced in the RCC4-VHL line by at least fivefold (*P *= 0.05) (Additional file 4), in keeping with targeting of ISCU1/2 by increased miR-210 expression. Consistent with this finding, a miR-210 mimic transfected into RCC4 + VHL cells to increase miR-210 levels did markedly decrease the mRNA expression of ISCU1/2 (threefold; *P *= 0.05) (Additional file 5a). Likewise, a miR-210 antagomir transfected into RCC4-VHL cells to decrease miR-210 levels markedly increased the mRNA expression of ISCU1/2 (twofold; *P *= 0.05) (Additional file 5b).

**Figure 10:**
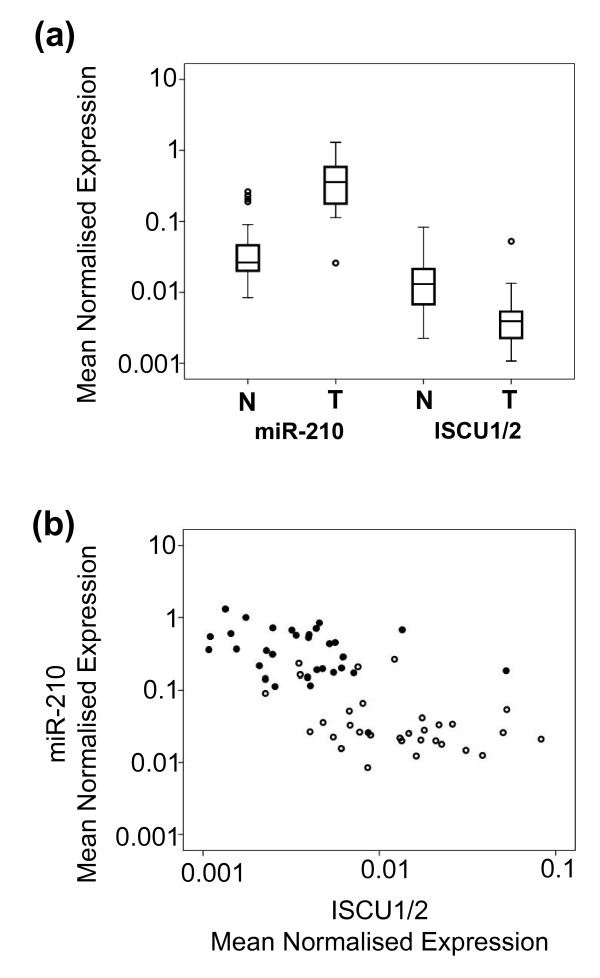
**Expression of ISCU1/2 is negatively correlated with miR-210 expression**. Measurements of ISCU1/2 and miR-210 expression were determined by qPCR and normalised to β-actin or an average of RNU43 and RNU48 expression. qPCR for each sample was performed in triplicate. **(a) **ISCU1/2 mRNA level is decreased in cc RCC tumours (T) compared to normal adjacent tissue (N) (*n *= 31), whereas the inverse expression pattern is observed for miR-210 (*P *≤ 0.0001). **(b) **A significant inverse correlation is observed between the miR-210 and ISCU1/2 expression level in both normal and tumour tissue (*r *= -0.679, *P *≤ 0.0001) (*n *= 62). Shaded circles indicate tumour tissue, and open circles represent adjacent normal tissue. Data are presented on log_10 _scales.

We therefore studied ISCU1/2 mRNA expression levels in the panel of renal cancers and found that, as previously shown, miR-210 expression is significantly upregulated in tumour tissue compared to normal adjacent tissue (*P *≤ 0.0001), whereas an inverse expression pattern is observed for ISCU1/2 mRNA (*P *≤ 0.0001) (Figure 10a). A striking negative correlation of miR-210 expression with ISCU1/2 mRNA in cc RCC tissue was observed, and, interestingly, this correlation was also observed in normal tissue (*r *= -0.679; *P *≤ 0.0001) (Figure 10b). This suggests that miR-210 may be involved in the physiological regulation of ISCU1/2 expression levels in normal tissues, as well as in tumours, and may contribute to the anaerobic pattern of respiration seen in renal (and other) tumours [63]. The levels of ISCU1/2 in renal cancers did not correlate with patient outcome, indicating that the influence of miR-210 level on patient survival is not mediated solely via ISCU1/2 regulation and that other miR-210 targets contribute to tumour aggression.

## Conclusions

In this study, we have identified miRNAs which are regulated by VHL in cell culture. For some of these miRNAs, the regulation was HIF-dependent and for others it was HIF-independent. This pattern of regulation was also seen in cc RCC for some of these miRNAs when compared with normal adjacent renal tissue (miR-210, miR-155, let-7i and miR-18a). The level of one HIF- and VHL-regulated miRNA, miR-210, showed marked increases in expression in the renal cancer tissue, and expression levels were correlated inversely with patient survival. The enhanced levels of miR-210 in renal cancer are likely to be mediated by unrestrained HIF activity, independent of hypoxia. It suggests that the association that has been seen in other cancers between miR-210 levels and patient survival is not solely because it is a marker of tumour hypoxia, but because it influences tumour behaviour through alterations in gene expression. miR-210 levels also showed a correlation with a HIF-regulated mRNA, CAIX, and with the presence of VHL mutation or promoter methylation. However, some tumours without evidence of VHL inactivation also had elevated miR-210 and CAIX levels, indicating the likely operation of other mechanisms of HIF activation. We also found a strong inverse correlation between miR-210 levels and mRNA expression of a miR-210 target gene, ISCU1/2, which may contribute to the repression of mitochondrial proteins by VHL and to the anaerobic pattern of respiration seen in renal (and other) tumours.

## List of abbreviations

CAIX: Carbonic anhydrase IX; cc RCC: clear cell renal cell carcinoma; DMOG: dimethyloxalylglycine; HIF: hypoxia-inducible factor; ISCU1/2: Iron-sulfur cluster assembly protein; miRNA: microRNA; MSP: methylation specific PCR; qPCR: relative quantitation real-time PCR; RCC: renal cell carcinoma; siRNA: small interfering RNA; TNM: Tumour Node Metastasis; VHL: von Hippel-Lindau.

## Competing interests

JMG is an inventor on a patent seeking to utilise miRNA expression as a prognostic marker in cancer. MZM is an inventor on a patent (pending) for technology that exploits differential miRNA expression to regulate the expression of therapeutic transgenes.

## Authors' contributions

CSN performed the experiments, undertook statistical analysis and drafted the paper.

MZM supervised the experiments, participated in the design of the study and drafted the paper. LHR designed and analysed the VHL molecular genetic studies. MBVdH coordinated and analysed the microarray experiments. JMG designed the study, supervised the experiments and drafted the paper. All authors read and approved the final manuscript.

## Pre-publication history

The pre-publication history for this paper can be accessed here:

http://www.biomedcentral.com/1741-7015/8/64/prepub

## Supplementary Material

Additional File 1 **Protein expression of HIF-1α and HIF-2α in RCC4 ± VHL cells and RCC4 + VHL cells treated with DMOG **Immunoblot of cell extract obtained from RCC4 ± VHL cells and RCC4 + VHL cells following DMOG treatment (1 mM, 24 hours). More HIF-1α and HIF-2α expression is seen in the RCC4-VHL cells and the DMOG treated RCC4+VHL cells compared to RCC4 + VHL cells. Click here for file

Additional File 2 **CAIX expression in RCC4 ± VHL cells and RCC4 + VHL cells treated with DMOG**. Measurements of CAIX mRNA expression was determined by qPCR and normalised to β-actin mRNA expression. qPCR for each sample was performed in triplicate. Expression of CAIX was found to be increased in RCC4-VHL cells and RCC4 + VHL cells treated with DMOG (1 mM, 24 hours) compared to RCC4 + VHL cells (*P *= 0.05). Click here for file

Additional File 3 **Table S1. Summary of genetic changes found in VHL**. The mutation nomenclature is in accordance with HGVS (Human Genome Variation Society) recommendations [31]. Nucleotide numbering is in accordance with GenBank mRNA sequence [GenBank:L15409] with the A of the first initiator ATG being 1. Previous descriptions of mutations were ascertained from http://www.umd.be[64], [33] and [65]. N* indicates that precise mutation has not been reported; however, similar mutations involving the same codon have been described. Key: del-deletion, ins-insertion, MS-missense, FS- frameshift, N-nonsense, spl-splice error. Click here for file

Additional File 4 **Expression of ISCU1/2 in RCC4+/-VHL cells **Measurements of ISCU1/2 expression was determined by qPCR and normalised to β-actin mRNA expression. qPCR for each sample was performed in triplicate. **(a) **ISCU1/2 mRNA level is decreased in RCC4-VHL cells compared to RCC4 + VHL cells (*P *= 0.05). Click here for file

Additional File 5 **Expression of ISCU1/2 following miR-210 overexpression and repression **Measurements of ISCU1/2 expression were determined by qPCR and normalised to β-actin mRNA expression. qPCR for each sample was performed in triplicate. **(a) **ISCU1/2 expression level was decreased in RCC4+VHL cells transfected with a miR-210 mimic compared to untransfected RCC4 + VHL cells and RCC4 + VHL cells transfected with a negative control (*P *= 0.05). **(b) **ISCU1/2 expression level was increased in RCC4-VHL cells transfected with a miR-210 antagomir compared to untransfected RCC4-VHL cells and RCC4-VHL cells transfected with a negative control (*P *= 0.05). Click here for file
