# Supplementary figures and images for: The VHL-dependent regulation of microRNAs in renal cancer
Source: BMC Med. 2010 Oct 21;8:64. doi: 10.1186/1741-7015-8-64 (PMC2978113; doi:10.1186/1741-7015-8-64)

## Slide 1
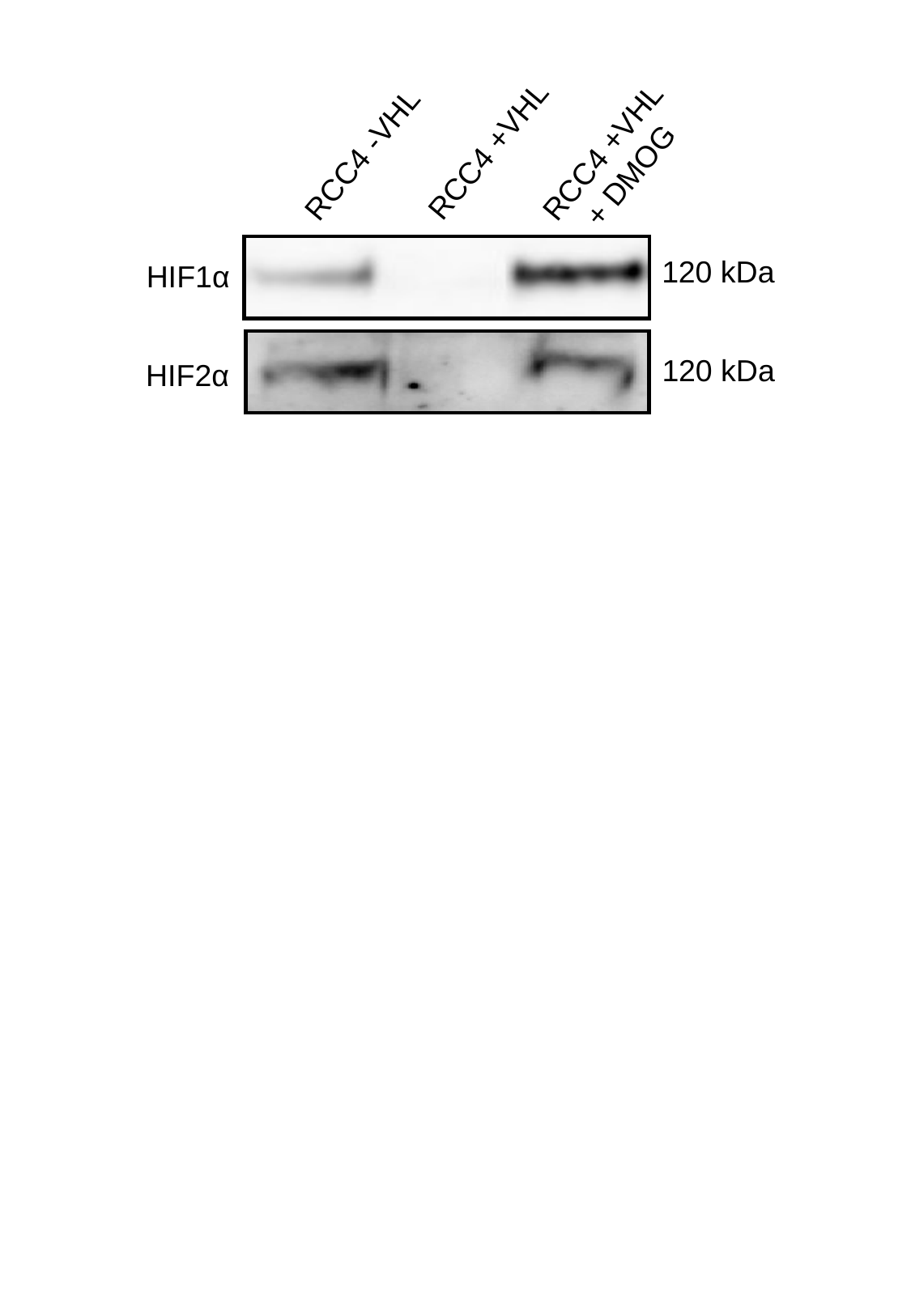

RCC4 +VHL
+ DMOG
RCC4 +VHL
RCC4 -VHL
120 kDa
HIF1α
120 kDa
HIF2α

Supplement: Additional File 1 — Protein expression of HIF-1α and HIF-2α in RCC4 ± VHL cells and RCC4 + VHL cells treated with DMOG Immunoblot of cell extract obtained from RCC4 ± VHL cells and RCC4 + VHL cells following DMOG treatment (1 mM, 24 hours). More HIF-1α and HIF-2α expression is seen in the RCC4-VHL cells and the DMOG treated RCC4+VHL cells compared to RCC4 + VHL cells. [file 1741-7015-8-64-S1.PPT]

## Slide 1
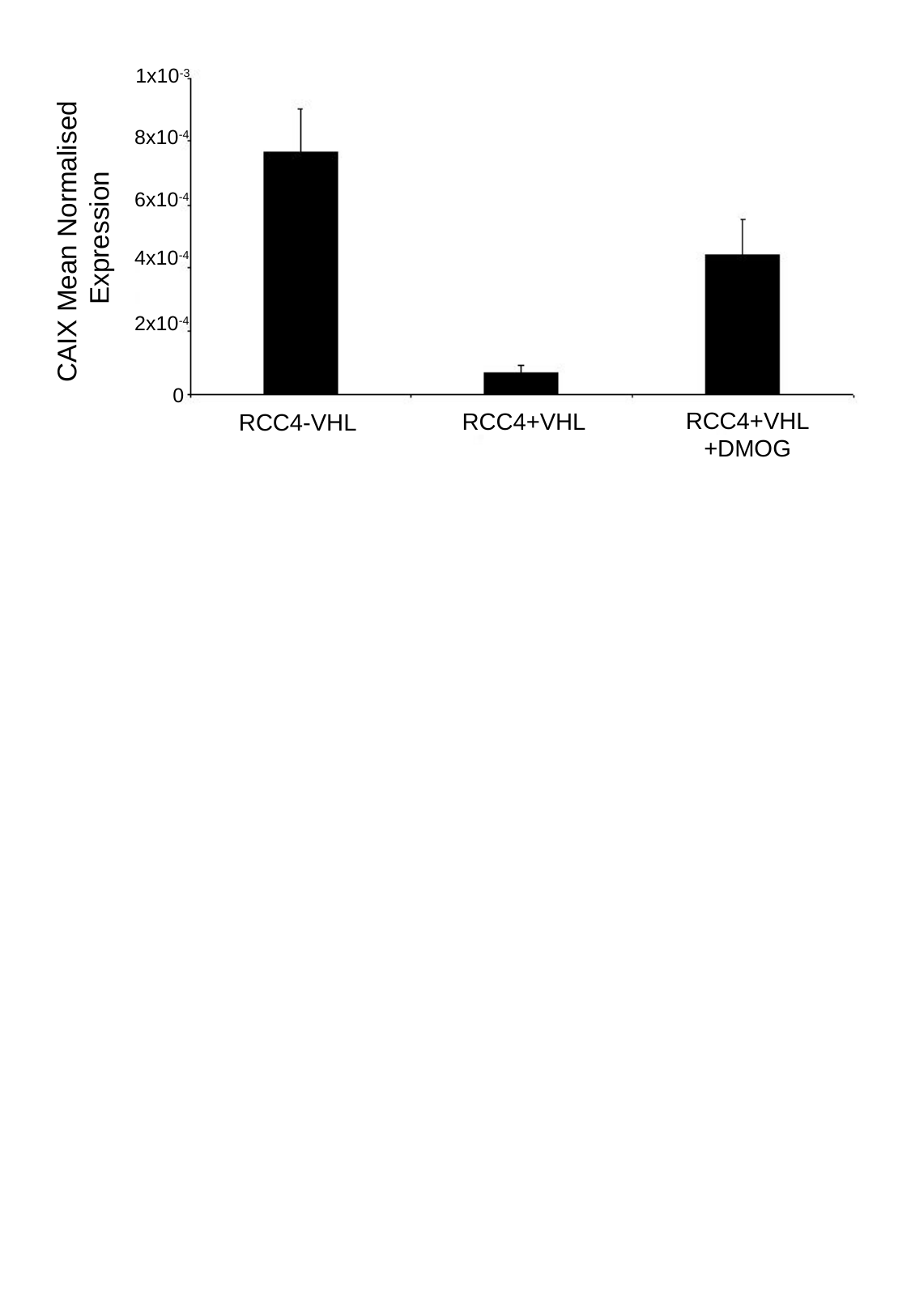

1x10-3
8x10-4
6x10-4
CAIX Mean Normalised
Expression
4x10-4
2x10-4
0
RCC4+VHL
+DMOG
RCC4+VHL
RCC4-VHL

Supplement: Additional File 2 — CAIX expression in RCC4 ± VHL cells and RCC4 + VHL cells treated with DMOG. Measurements of CAIX mRNA expression was determined by qPCR and normalised to β-actin mRNA expression. qPCR for each sample was performed in triplicate. Expression of CAIX was found to be increased in RCC4-VHL cells and RCC4 + VHL cells treated with DMOG (1 mM, 24 hours) compared to RCC4 + VHL cells (P = 0.05). [file 1741-7015-8-64-S2.PPT]

## Slide 1
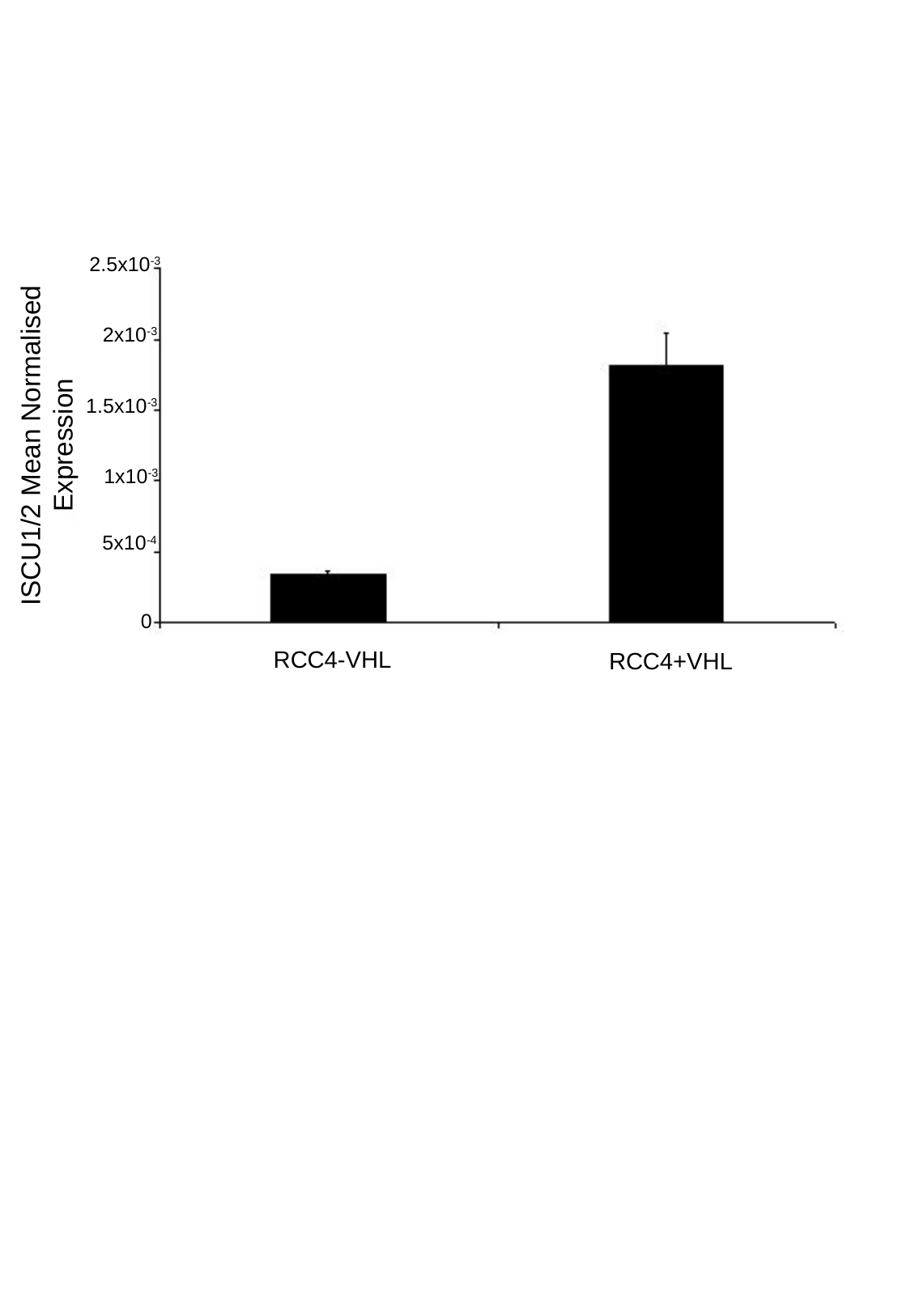

2.5x10-3
2x10-3
1.5x10-3
ISCU1/2 Mean Normalised Expression
1x10-3
5x10-4
0
RCC4-VHL
RCC4+VHL

Supplement: Additional File 4 — Expression of ISCU1/2 in RCC4+/-VHL cells Measurements of ISCU1/2 expression was determined by qPCR and normalised to β-actin mRNA expression. qPCR for each sample was performed in triplicate. (a) ISCU1/2 mRNA level is decreased in RCC4-VHL cells compared to RCC4 + VHL cells (P = 0.05). [file 1741-7015-8-64-S4.PPT]
