# Supplementary material for: The VHL-dependent regulation of microRNAs in renal cancer
Source: BMC Med. 2010 Oct 21;8:64. doi: 10.1186/1741-7015-8-64 (PMC2978113; doi:10.1186/1741-7015-8-64)
Supplement: Additional File 3 — Table S1. Summary of genetic changes found in VHL. The mutation nomenclature is in accordance with HGVS (Human Genome Variation Society) recommendations [31]. Nucleotide numbering is in accordance with GenBank mRNA sequence [GenBank:L15409] with the A of the first initiator ATG being 1. Previous descriptions of mutations were ascertained from http://www.umd.be[64], [33] and [65]. N* indicates that precise mutation has not been reported; however, similar mutations involving the same codon have been described. Key: del-deletion, ins-insertion, MS-missense, FS- frameshift, N-nonsense, spl-splice error. [file 1741-7015-8-64-S3.DOC]

| **Patient** | **Methylation** | **Exon** | **mRNA change** | **Codon/amino acid change** | **Mutation type** | **Previously found** |
| --- | --- | --- | --- | --- | --- | --- |
| 1 | - | - | - | - | - |  |
| 2 | - | - | - | - | - |  |
| 3 | - | - | - | - | - |  |
| 4 | - | 1 | c.238_240 delins CCC | p.S80P | del/ins | No |
| 5 | - | 2 | c.341-1_341-14del | p.? | Spl | No* |
| 6 | - | - | - | - | - |  |
| 7 | - | 3 | c.481 C>T | p.R161X | N | Yes |
| 8 | - | - | - | - | - |  |
| 9 | Yes | - | - | - | - |  |
| 10 | - | - | - | - | - |  |
| 11 | - | 3 | c.571 del | p.H191T fsX11 | FS | Yes |
| 12 | - | 1 | c.209_215 del | p.E70A fsX87 | FS | No* |
| 13 | - | - | - | - | - |  |
| 14 | - | - | - | - | - |  |
| 15 | - | - | - | - | - |  |
| 16 | - | 3 | c.543_554 del | p.R182_Y185 del | Del | No |
| 17 | - | - | - | - | - |  |
| 18 | - | - | - | - | - |  |
| 19 | - | 3 | c.510_514 dup | p.E173S fsX31 | FS | No |
| 20 | - | - | - | - | - |  |
| 21 | Yes | 1 | c.194 C>A | p.S65X | N | Yes |
| 22 | - | - | - | - | - |  |
| 23 | - | - | - | - | - |  |
| 24 | - | 2 | c.406T>G | p.F136V | MS | Yes |
| 25 | - | 2 | c.341-1_341-4 del | p.? | Spl | No* |
| 26 | - | 3 | c.472_474 del | L158del fsX15 | FS | Yes |
| 27 | Yes | - | - | - | - |  |
| 28 | - | 2 | c.451 dup | p.I151N fsX23 | FS | Yes |
| 29 | - | - | - | - | - |  |
| 30 | - | 1 | c.120_162 del | p.E41R fsX12 | FS | No |
| 31 | - | - | c.341-2 A>T | p.? | Spl | No* |
