# Supplementary material for: The VHL-dependent regulation of microRNAs in renal cancer
Source: BMC Med. 2010 Oct 21;8:64. doi: 10.1186/1741-7015-8-64 (PMC2978113; doi:10.1186/1741-7015-8-64)
Supplement: Additional File 5 — Expression of ISCU1/2 following miR-210 overexpression and repression Measurements of ISCU1/2 expression were determined by qPCR and normalised to β-actin mRNA expression. qPCR for each sample was performed in triplicate. (a) ISCU1/2 expression level was decreased in RCC4+VHL cells transfected with a miR-210 mimic compared to untransfected RCC4 + VHL cells and RCC4 + VHL cells transfected with a negative control (P = 0.05). (b) ISCU1/2 expression level was increased in RCC4-VHL cells transfected with a miR-210 antagomir compared to untransfected RCC4-VHL cells and RCC4-VHL cells transfected with a negative control (P = 0.05). [file 1741-7015-8-64-S5.PPT]

## Slide 1
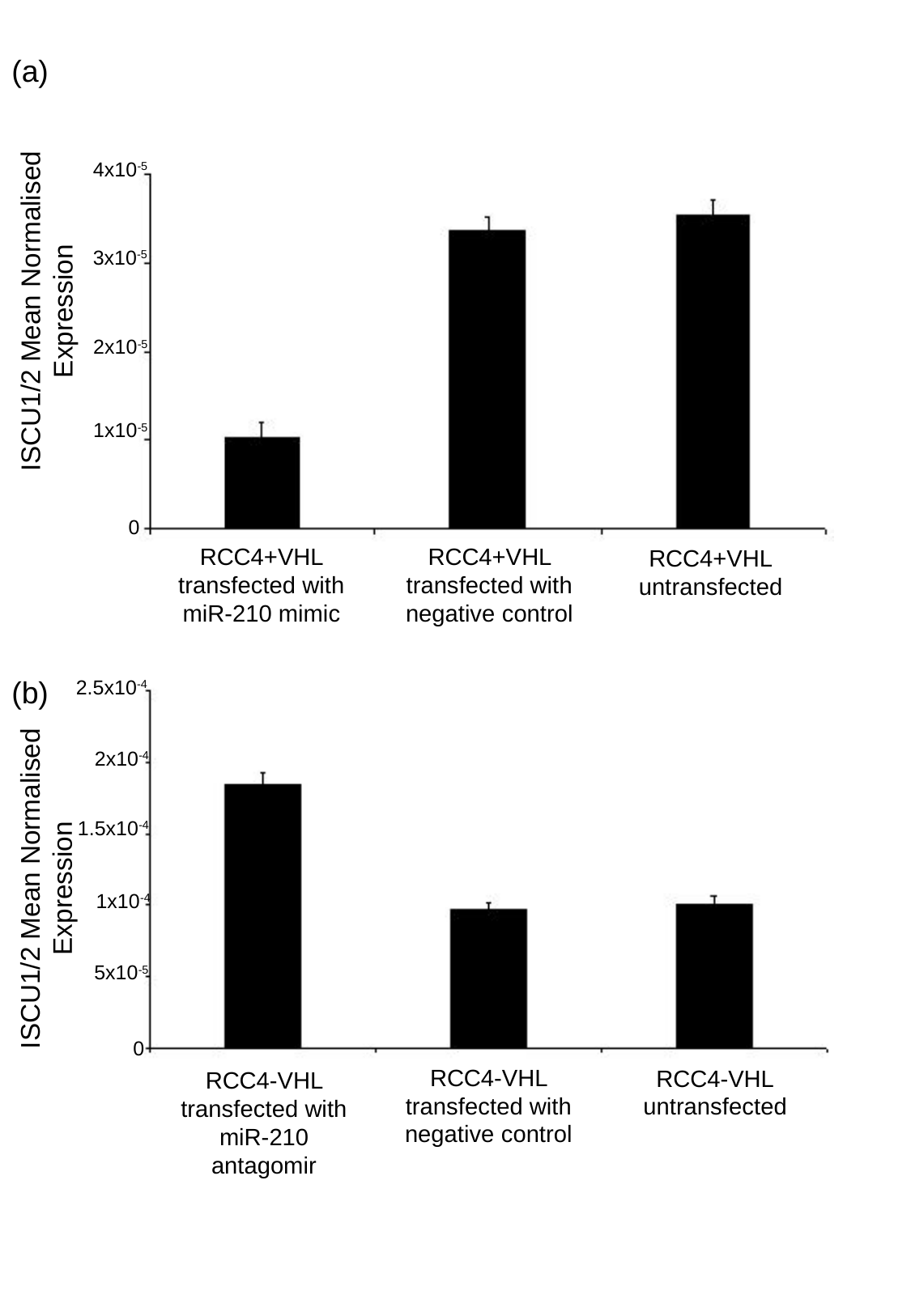

(a)
4x10^-5^
3x10^-5^
ISCU1/2 Mean Normalised Expression
2x10^-5^
1x10^-5^
0
RCC4+VHL transfected with miR-210 mimic
RCC4+VHL transfected with negative control
RCC4+VHL
untransfected
(b)
2.5x10^-4^
2x10^-4^
1.5x10^-4^
ISCU1/2 Mean Normalised Expression
1x10^-4^
5x10^-5^
0
RCC4-VHL transfected with negative control
RCC4-VHL
untransfected
RCC4-VHL transfected with miR-210 antagomir
